# Abuse potential assessment of propofol by its subjective effects after sedation

**DOI:** 10.12669/pjms.306.5811

**Published:** 2014

**Authors:** Aysu Hayriye Tezcan, Dilsen Hatice Ornek, Onur Ozlu, Mustafa Baydar, Nurcan Yavuz, Nihal Gokbulut Ozaslan, Kevser Dilek, Aylin Keske

**Affiliations:** 1 Dr. Aysu Hayriye Tezcan, Department of Anesthesia and Reanimation, Ankara Numune Education and Research Hospital, Ankara, Turkey.; 2 Dr. Dilsen Hatice Ornek, Department of Anesthesia and Reanimation, Ankara Numune Education and Research Hospital, Ankara, Turkey.; 3 Prof. Dr. Onur Ozlu, Department of Anesthesia and Reanimation, Duzce University Faculty of Medicine, Duzce, Turkey.; 4 Dr. Mustafa Baydar, Department of Anesthesia and Reanimation, Ankara Numune Education and Research Hospital, Ankara, Turkey.; 5 Dr. Nurcan Yavuz, Department of Anesthesia and Reanimation, Ankara Numune Education and Research Hospital, Ankara, Turkey.; 6 Dr. Nihal Gokbulut Ozaslan, Department of Anesthesia and Reanimation, Ankara Numune Education and Research Hospital, Ankara, Turkey.; 7 Dr. Kevser Dilek, Department of Anesthesia and Reanimation, Ankara Numune Education and Research Hospital, Ankara, Turkey.; 8 Dr. Aylin Keske, Department of Anesthesia and Reanimation, Ankara Numune Education and Research Hospital, Ankara, Turkey.

**Keywords:** Abuse potential, Euphoric effect, Propofol, Sedation

## Abstract

***Objective:*** In this study, we examined the euphoric effect of propofol and its high satisfaction ratio regarding its liability to be abused, particularly in painless procedures, such as colonoscopy.

***Methods: ***Fifty subjects aged between 18 and 65 years who fulfilled the criteria for ASA 1-2 and were prepared for colonoscopy were enrolled into this study. For intravenous sedation induction, 2 mg/kg propofol was used, and additional injections were administered according to BIS values. After colonoscopy, the subjects were taken to a recovery room and observed for 30 minutes. Patients were interviewed with the modified Brice questionnare regarding the incidence and the content of dreams. A 5-point Likert scale was used to classify their dreams, and the content of the dreams was also recorded. To assess the subjective effects of propofol, the patients were asked to use the Hall and Van der Castle emotion scale; their biological states were also assessed. The patients’ feelings regarding propofol were each rated as absent or present. We used the Morphine-Benzedrine Group scale to measure the euphoric effects of propofol. At the end of the study, subjects scored their satisfaction on a five-point scale.

***Results: ***There were no statistically significant differences in sex age, weight, propofol dose, or satisfaction ratio (p>0.05) in the groups, although male patients received a higher dose of propofol and had higher satisfaction ratio. Patients reported no residual after-effects. The incidence of dreaming was 42%. There was no statistically significant difference in dreaming between the sexes, but male patients had a higher dreaming ratio. Dreamers received higher propofol doses and had a higher satisfaction ratio (p>0.05). All dreamers reported happy dreams regarding daily life, and their mean MBG score was 10.5. There was no correlation between MBG scores and propofol doses (r= -0.044, p= 0.761).

***Conclusions: ***We conclude that propofol functions as a reward; that patients enjoy its acute effects; and that no residual after-effects should arise. We suggest that propofol may carry potential for abuse, and further abuse liability testing is indicated.

## INTRODUCTION

Propofol is a potent anesthetic drug that rapidly causes sedation and unconciousness when intravenously injected.^1^^-^^5^ Unconciousness occurs 30 seconds after the injection of 1.5-2.5 mg/kg propofol, and if not infusion is initiated, the drug’s effects last 5-10 minutes.^1^^,^^2^^,^^4^ The administration of 2.5mg/kg propofol results in a 25-40% decrease in blood pressure, blunting the respiratory drive and relaxing the oropharyngeal muscle tone towards the development of apnea.^1^^,^^2^^,^^4^ A single injection of propofol can cause apnea, hypoxia, and death without proper management by a medical professional. These effects enhance the potential danger of propofol and highlight its possible lethality if administered by an inexperienced personnel or even oneself.^1^^,^^2^^,^^4^

In vivo studies have subsequently supported the potential for abusing propofol. Propofol is an activator of the GABA-A receptor, similar to other frequently abused drugs (e.g., alcohol, benzodiazepines, and barbiturates).^4^ In another study, the authors showed that propofol increases glutamatergic excitatory synaptic transmission and discharges dopamine neurons in the ventral tegmental area, potentially contributing to the development of propofol abuse.^5^ Subanesthetic and anesthetic doses of propofol increase the concentration of dopamine in the nucleus accumbens, as previously described among such commonly abused drugs as opiates, cocaine, and amphetamine.^6^^,^^7^

Propofol’s fast recovery, amnestic properties, and safety profiles are among the important advantages that have led to the high use of this drug for sedation in ambulatory settings.^1^^,^^8^ Patient satisfaction may increase due to the anxiolytic and mood-altering effects of the drug. Although it has many ideal aspects, there have been several cases of drug abuse and addiction.^9^ We know that these euphoric effects are critical draws for patients who abuse drugs and may be assessed with the MBG scale.^10^ To the best of our knowledge, not enough clinical trial exists investigating the potential for patient abuse of propofol.

In this study, we examined the potential for abuse of propofol based on its euphoric effects, as assessed by the MBG scale and patient satisfaction ratio in painless procedures, such as colonoscopies.

## METHODS

This prospective study was approved by the Ankara Numune Education and Research Hospital Institutional Review Board. Written informed consent from each patient was obtained before the study. Fifty patients aged 18-65 years fulfilling the ASA 1-2 criteria who were prepared for colonoscopy were selected for this study. Subjects received only propofol sedation during the colonoscopy. None of the subjects had prior experience with alcohol, stimulants or any abused drugs. Subjects with a history of any neuropsychiatric disorders, such as depression, were excluded, and a basic medical history was included. Subjects had been instructed not to eat food or drink 6 hours before the procedure. Blood tests were performed on subjects to determine their normal liver and kidney functions before the procedure.

Subjects were questioned about their preoperative anxiety using a ten-point scale (0 point=not anxious; 10 points=very anxious) and about their dreaming habits using a 3-point scale (0 points=rare; 1 point=once a month; 2 points=once a week; 3 points=almost every night).

Non-invasive measurements of heart rate, electrocardiogram, peripheral oxygen saturation, systolic and diastolic blood pressure, and BIS monitoring were initiated at the beginning of the colonoscopy before sedation had been started. After recording the first values of the subject, a venous cannulation was inserted into subjects’ forearms, and the sedation was initiated. Noninvasive physiologic measurements were assessed before sedation and during the sedation with ten minutes intervals. For the induction of intravenous sedation, 2mg/kg propofol was used, and additional injections (0.5mg/kg per injection) were administered according to heart rate or BIS value increases. We avoided continuous infusions to minimize the drug dosage and to demonstrate its euphoric effects in low dosages.

After colonoscopy, all subjects were taken to a recovery room and observed for 30 minutes. In the recovery room, subjects were interviewed about effects of the anesthesia. None of the subjects learned the name or any other information of the anesthetic drug. All questionnaires were completed 15 minutes after patient recovery. Recovery was defined as the patient’s orientation to time and place.

Patients were interviewed with the modified Brice questionnare about the incidence and the content of their dreams.^11^ Patients were asked “What was the last thing you remembered before going to sleep?”, “What was the first thing you remembered when you woke up?”, “Can you recall anything between?” and “Did you have any dreams during your anesthesia?”. If dreaming was reported, a 5-point Likert (memorability, emotional content, visual vividness, emotional intensity, strangeness) scale was used to classify the dreams. Further, the content of the dreams was also recorded.

To assess the subjective effects of propofol after the procedure, patients were asked whether they were experiencing any of the five feelings found on the Hall & Vander Castle emotion scale (angry, apprehensive, happy, sad, or confused).^11^^,^^12^ They were also asked about their biological states (sick, hungry, high, sedated, dizzy, light-hearted),^11^^,^^12^ with each feeling being rated as absent or present.

The most critical item in the assessment of potential abuse involves the subjective effects of the drug, which may include emotions, perceptions, and moods.^10^ These questionnaires attempted to measure the presence or absence of the drug’s effects and/or mood changes. A critical item in this regard is the assessment of subjects liking certain behaviors in euphoric states facilitated by the drug in question; one of the most common questionnare subscales in this regard is taken from the addiction research center inventory (ARCI).^9^^,^^10^^,^^13^ The most frequently used scales used to study the effects of potential drug abuse include the Morphine-Benzedrine Group (MBG; an index of euphoria), the Pentobarbital-Chlorpromazine-Alcohol Group (PCAG; an index of sedation), and the Lysergic Acid Diethylamide Group (LSD; an index of dysphoria or somatic discomfort). Increases in the MBG scale (euphoria scale) are associated with significant potential for abuse. The addiction research center inventory (ARCI) is a true/false questionnaire designed to differentiate among classes of psychoactive drugs (1 point is given to “no”, and 0 points, to “yes”). The ARCI includes 49 items on five different scales. We used the Morphine-Benzedrine Group scale to measure the euphoric effects of propofol. The MBG scale can be expressed as a score from 0-16. One point is awarded for each true response in items 1-10 and 12-16, and one point is given for each false response in the reverse-scored item.^9^^,^^10^

At the end of the study, subjects scored their satisfaction on a five-point scale (0 “none”; 5 “very high satisfaction”).

***Statistics: ***In this study, the SPSS 20 software package was used for the statistical analysis. To detect an effect size of 0.4 at alpha error of 0.05 and statistical power of 0.80, 50 participants were required.  Wilcoxon and Mann-Whitney U tests were performed, and a mean comparison was performed to analyze the sex and age characteristics of the subjects. Correlation analysis was performed to investigate whether age and sex were related to MBG scores. The results of the analysis were considered to be significant when p<0.05. 

## RESULTS

Data collection was completed for 50 patients. All of the patients received only one agent (propofol) for sedation. Patients’ demographic data can be seen in Table-I. The duration of the procedure varied between 15 and 75 minutes (mean: 27.1 min). Overall, 12 (24%) patients had no comorbidities; 10 (20%) had hypertension; 9 (18%) had diabetes mellitus; 7 (14%) had thyroidal diseases that were under control; 3 (6%) had coronary artery disease; 3 (6%) had asthma; 2 (4%) had malignancy; and 4 (8%) had other unimportant diseases. There were no statistically significant differences between the patient groups in sex, age, weight, propofol dose, or satisfaction ratio (p>0.05), although male patients did receive a higher dose of propofol and had a higher satisfaction ratio. The incidence of dreaming was 42%. There was no statistically significant difference regarding dreaming between sex, but male patients did have a higher dreaming ratio. Although there was no statistically significant difference between dreamers and non-dreamers regarding their propofol doses or satisfaction ratios, dreamers received higher propofol doses and had a higher satisfaction ratio (p>0.05)(Table-II). The median and interquartile ranges of the Likert scale were memorability 2.9 (1-5), emotional content 2.9 (0-5), visual vividness 2.5 (0-5), emotional intensity 2.6 (0-5), and strangeness 0 (0-5). All dreamers reported happy dreams about their daily lives, and the mean MBG score was 10.5. There was no significant difference according to sex or age, but dreamers had significantly higher MBG scores (p<0.05) (Table-II, III). There was no correlation between the MBG score and the propofol dose (r=-0.044, p=0.761). The results of the emotional and bodily state questionnaires are shown in Table-IV. Overall, 80% of the patients reported happiness, 40% of which felt light-hearted. There were no dangerous changes in the physiologic measures of the patients using propofol. After propofol exposure, the heart rate and peripheric oxygen saturation stayed stable, and only a minimal decrease in mean blood pressure was observed. Only one patient reported a residual effect, in this case, nausea. The mean patient recovery time was 5.7 (SD:2.1) minutes.

## DISCUSSION

Propofol has become increasingly abused because it is easily accessible, has a rapid onset of action, and has an ultra-short duration of action without any long-term obvious residual side effects.^2^^,^^4^^,^^7^ The first case report on propofol dependence focused on an anesthesiologist. Although the case was not extraordinary, it was the first in the field to detail the reasons behind the anesthesiologist’s preference for propofol. He had also tried midazolam and fentanyl injections, but propofol was easily accessed, was ultra-short-acting, and had no side effects. Although he first attempted to use propofol for stress relief, he soon experienced an overwhelming compulsion and craving to use it again.^6^ The majority of cases of propofol abuse involve the use of the drug for non-anesthetic purposes, such as stress relief, insomnia relief and euphoria.^2^^,^^3^^,^^12^^,^^14^

**Table-I:** Demographics.

	***Total (n=50)***	***Male (n=17)***	***Female (n=33)***	***p value***
Age (yr)	47.2±16.3	45.1±16.0	48.3±16.6	0.47
Weight (kg)	74.8±15.3	74.2±14.6	75.1±15.9	0.81
Propofol dose(mg)	279.6±135.9	324.1±125.6	256.7±137.0	0.07
MBG Scale(0-16)	10.5± 5.3	10.9±3.2	10.3±3.4	0.61
Dreamers (%)	21(42)	9(52.9)	12(36.4)	0.41
Satisfaction Ratio (0-5)	4.5±0.7	4.6±0.5	4.5±0.8	0.49

**Table-II:** Difference between dreamers and non-dreamers

	***Total (n=50)***	***Dreamers (n=21)***	***Non-dreamers (n=29)***	***p value***
Age (yr)	47.2±16.3	47.2±13.3	47.2±18.4	0.86
Weight (kg)	74.8±15.3	75.6±15.7	72.2±15.3	0.76
Propofol dose(mg)	279.6±135.9	310.5±152.3	257.2±120.4	0.28
MBG Scale(0-16)	10.5±5.3	11.9±2.6	9.4±3.5	0.00
Satisfaction Ratio (0-5)	4.5±0.7	4.7±0.5	4.4±0.8	0.30

**Table-III:** MBG Score and age

	***Age***	***N***	***Mean (min.-max.)***	***p***
MBG Score	20-30	8	9,8(3-15)	0.564
	31-40	11	11,6(5-15)	
	41-50	9	9,9(3-15)	
	51+	22	10,4(3-14)	

**Table-IV:** Number of patients reporting the presence of each emotion or bodily state.

	***N***	***%***
Angry	1	2
Happy	40	80
Sad	3	6
Apprehensive	2	4
Confused	8	16
Hungry	12	24
Sick	4	8
High	13	26
Sedated	6	12
Dizzy	9	18
Light-hearted	20	40

After painful postoperative conditions, patients may have unpleasant emotions that could affect their evaluation of their likelihood of abusing propofol.^6^^,^^12^ Therefore, this study was performed on patients submitting to a colonoscopy with minimal or no pain. Despite the widespread use of this drug for anesthesia, few cases of abuse have been observed.^14^^-^^16^ Other authors have expressed that being aware of propofol’s use in anesthesia and subsequent, repeated pleasurable effects is the most important contributing factor to developing any propofol dependence. 

For propofol, psychological dependence is more common than physical dependence because propofol causes euphoria, stress relief, sexual fantasies and dreams, and sexual disinhibition.^2^^,^^7^^,^^15^^,^^16^ These effects of propofol lead to drug-craving and loss of control over the amount and frequency of drug injections, as well as the continued use of propofol regardless of any adverse consequences. In our study, subjects did not report any sexual dreams, and all indicated very pleasant situations in their dreams about daily life. We speculate that these happy dreams may contribute to propofol’s euphoric effects.

In an attempt to determine the subjective and emotional effects of the drug, we recorded the subjects’ dreams, as well as the emotional content of the dreams, the MBG scale, modified emotional and bodily state scale, and satisfaction ratio. Mu opioid agonists (morphine, heroin) are among the highly abused drugs that produce high MBG scale scores.^10^ Although another study on marijuana found an MBG score of 4.7, in this study on propofol, the MBG score was calculated to be 10.5. These values indicate that propofol has strong euphoric effects. In a previous study performed on patients receiving gastric endoscopies, the MBG score was 6.3 after propofol exposure, a lower value than found here.^9^ We postulate that colonoscopies are longer procedures than gastric endoscopies, resulting in patients’ receiving higher doses of propofol during these longer procedures and potentially explaining the difference in scores between the studies. In propofol addiction cases, the induction of a comfortable sleep was another reason for propofol’s preference; therefore, we aimed to evaluate the relationships among dreaming, MBG scales and satisfaction ratios in this study. No significant difference was found, but dreamers did receive higher propofol doses and produced higher satisfaction ratio. We observed that all the dreams were pleasant and that the dreamers woke up happy after the procedure. 

The MBG scores showed a statistically significant difference between dreamers and non-dreamers. The dreamers’ MBG score was 11.9, whereas the score of non-dreamers was 9.4 (p<0.05). Based on this information, pleasant dreaming may contribute to this drug’s potential for abuse. After completing a modified emotional scale questionnaire, all patients reported generally positive emotions, such as happy, light hearted, and high. After the procedure, the patient satisfaction ratio was extremely high (mean:4.5). Based on the accumulated data, we conclude that propofol has pleasant subjective effects that may reinforce and/or cause its abuse. Studies on healthy volunteers taking propofol support our assessment of the effects of this drug.^3^^,^^11^^,^^12^^,^^17^ Another important point about propofol preference is its minimal residual effect profile.^2^^,^^5^^,^^10^^,^^18^ In this study, only one patient reported any residual effects, which in this case was nausea. In this study, we only investigated the acute effects of the drug after a single exposure. Additional research is needed to improve our understanding of the addictive characteristics of propofol after repeated exposure.

The effect profiles of certain psychomotor stimulants, including cocaine and amphetamine, morphine, and heroin produce very similar subjective effect profiles. Both enhance a patient’s positive disposition and MBG scale scores, consistent with their high liability for being abused.^10^ Although propofol is widely used by millions of patients, related dependence cases are few in number. The authors believe that the reason behind this statistic is that most propofol users do not know the identity of their administered anesthetic.

The most dramatic and important outcome of recreational propofol abuse is the potential for death by unconscious state and apnea following its injection.^1^^,^^2^^,^^4^^,^^11^^,^^18^^-^^21^ A review reported on the number of propofol dependence/abuse human cases between 1992 to 2007, finding that 38 human cases resulted in fatality. Twelve of these deaths were medical professionals, and 9 were anesthesiologists.^16^

In conclusion, the present study found that propofol is an ideal sedative agent based upon its pleasant effects, rapid recovery time, inconsiderable residual effects, and few or lack of physiological changes. However, these properties make propofol an ideal drug for abuse when medical professionals, patients or lay persons with abusive tendencies discover its effects. In most hospitals, medical professionals and other staff members are at risk because propofol dispensing is not adequately controlled, making access to this anesthetic far too easy. Strict control of this drug will still not eradicate propofol abuse entirely, but if it is not designated as a controlled substance, its distribution may extend past hospitals, endangering thousands of lay persons. This important issue should be further evaluated by public health authorities in future studies.

## Authors Contribution:

**AHT:** Conceived, designed and performed the statistical analysis & editing of manuscript.

**AHT, MB, NY, NGO, KD, AK:** Collected data and wrote the manuscript.

**DHO, OO:** Reviewed and gave final approval of the manuscript.

**AHT:** Takes responsibility and is accountable for all aspects of the work in ensuring that questions related to the accuracy or integrity of any part of the work are appropriately investigated and resolved.

## References

[1] Marik PE (2004). Propofol: therapeutic indications and side effects. Curr Pharm Des.

[2] Sipe BW, Scheidler M, Baluyut A, Wright B (2007). A prospective safety study of a low-dose propofol sedation protocol for colonoscopy. Clin Gastroenterol Hepatol.

[3] Abad-Santos F, Gálvez-Múgica A, Santos A, Novalbos J, Gallego-Sandín S, Méndez P, et al (2003). Pharmacokinetics and Pharmacodynamics of a Single Bolus of Propofol 2% in Healthy Volunteers. J Clin Pharmacol.

[4] Krasowski MD, Harrison NL (1999). General Anaesthetic Actions On Ligand-Gated İon Channels. Cell Mol Life Sci.

[5] Li KY, Xiao C, Xiong M, Delphin E, Ye JH (2008). Nanomolar Propofol Stimulates Glutamate Transmission to Dopamine Neurons: A Possible Mechanism of Abuse Potential?. J Pharmacol Experimental Therapeutics.

[6] Follette J, Farley WJ (1992). Anesthesiologist Addicted to Propofol. Aneesthesiology.

[7] Pain L, Gobaille S, Schleef C, Aunis D, Oberling P (2002). In Vivo Dopamine Measurements in the Nucleus Accumbens After Nonanesthetic and Anesthetic Doses of Propofol in Rats. Anesth Analg.

[8] Zed PJ, Abu-Laban RB, Chan WW, Harrison DW (2007). Efficacy, safety and patient satisfaction of propofol for procedural sedation and analgesia in the emergency department: a prospective study. Canadian Assoc Emerg Physicians.

[9] Kim JH, Byun H, Kim JH (2013). Abuse potential of propofol used for sedation in gastric endoscopy and its correlation with subject characteristics. Korean J Anesthesiol.

[10] Steven B Karch (1998). Drug Abuse Handbook.

[11] Brandner B, Blagrove M, McCallum G, Bromley LM (1997). Dreams, images and emotions associated with propofol anaesthesia. Anaesthesia.

[12] Zacny JP, Lichtor JL, Thompson W, Apfelbaum JL (1993). Propofol at a Subanesthetic Dose May Have Abuse Potential in Healthy Volunteers. Anesth Analg.

[13] de Wit H, Griffiths RR (1991). Testing the abuse liability of anxiolytic and hypnotic drugs in humans. Drug Alcohol Dependence.

[14] Schneider U, Rada D, Rollnik JD, Passie T, Emrich HM (2001). Propofol dependency after treatment of tension headache. Addict Biol.

[15] Kirby RR, Colaw JM, Douglas MM (2009). Death from propofol: Accident, Suicide, or Murder?. Anesthesia Analgesia.

[16] Roussin A, Louis Montastruc J, Lapeyre-Mestre M (2007). Pharmacological and clinical evidences on the potential for abuse and dependence of propofol: A review of the literature. Fundam Clin Pharmacol.

[17] Zacny JP, Lichtor JL, Coalson DW, Finn RS, Uitvlugt AM, Glosten B (1992). Subjective And Psychomotor Effects Of Subanesthetic Doses Of Propofol In Healthy Volunteers. Anesthesiology.

[18] Fritz GA, Niemczyk WE (2002). Propofol Dependency In A Layperson. Anesthesiology.

[19] Kranioti EF, Mavroforou A, Mylonakis P, Michalodimitrakis M (2007). Lethal self administration of propofol (Diprivan) A case report and review of the literature. Forensic Sci Int.

[20] Klausz G, Pharm D, Rona K, Kristof I, Torő K (2009). Evaluation of a fatal propofol intoxication due to self administration. J Forensic Leg Med.

[21] Drummer OH (1992). A fatality due to propofol poisoning. J Forensic Sci.

